# Integrating the Central Sensitization Inventory (CSI) into Neuropelveological Practice: A Systematic Review of Endometriosis and Overlapping Pelvic Pain Syndromes

**DOI:** 10.3390/jcm15135187

**Published:** 2026-07-02

**Authors:** Piotr Lepka, Paulina Lepka, Marcin Jędryka

**Affiliations:** 1 Department of Oncological Gynecology, Lower Silesian Oncology, Pulmonology and Hematology Center, 53-413 Wroclaw, Poland; 2 Clinical Department of Gynaecological Oncology, Department of Oncology, Faculty of Medicine, Wroclaw Medical University, 50-367 Wroclaw, Poland; 3 Department of Otolaryngology, Head and Neck Surgery, 4th Military Teaching Hospital and Polyclinic in Wroclaw, 50-981 Wroclaw, Poland

**Keywords:** endometriosis, central sensitization, neuropelveology, LION procedure, chronic pelvic pain, Central Sensitization Inventory (CSI), nociplastic pain

## Abstract

**Background**: The surgical management of chronic pelvic pain (CPP), particularly in endometriosis, often focuses on lesion excision or nerve decompression. However, persistent pain frequently occurs despite “anatomical perfection,” suggesting central nervous system involvement. Neuropelveology faces a “surgical paradox” when dealing with central sensitization (CS), where peripheral interventions fail to address a systemic nociplastic state. **Methods**: This systematic review followed PRISMA guidelines and was registered in PROSPERO (CRD420261335008). A search across PubMed, Embase, and Cochrane (2010–2026) identified 71 relevant studies involving over 12,000 participants. **Results**: CS prevalence in the endometriosis population ranges from 11.3% to 58.2%, rising to 74.8% in specialized tertiary referral centers. The Central Sensitization Inventory (CSI) is a robust predictor of surgical failure; every one-point increase in preoperative CSI raises the risk of persistent pain (OR 1.02, *p* = 0.02). Objective markers, such as the collapse of Conditioned Pain Modulation (CPM), confirm that “high-sensitizers” (CSI ≥ 40) suffer from a systemic “software” failure of pain inhibition. **Conclusions**: We propose a paradigm shift in neuropelveology. In patients with high CSI scores (≥40), functional neuromodulation—specifically the LION procedure—should be prioritized over traditional nerve decompression to address the nociplastic nature of the pain.

## 1. Introduction

The management of endometriosis-associated chronic pelvic pain (CPP) has historically focused on the surgical excision of visible lesions. Furthermore, emerging research highlights the complex pathophysiological environment of the disease, where factors such as Vitamin D deficiency, receptor polymorphisms, and its role in modulating local inflammatory states and cellular proliferation are increasingly recognized [1]. However, the high rate of persistent or recurrent pain—often occurring in the absence of visible disease—suggests that peripheral pathology is only one component of a broader pain syndrome. Recent advancements in pain neuroscience have highlighted the role of Central Sensitization (CS), a state of nociplastic pain characterized by the amplification of neural signaling within the central nervous system [2]. Neuropelveology, a discipline at the intersection of neuroscience and pelvic surgery, has revolutionized our ability to address neural entrapments and infiltration. Yet, as established by the International Consensus Guidelines on Neuropelveology (2026) [3], surgical success depends on a precise pretherapeutic diagnosis that distinguishes between neurogenic, visceral, and central pain drivers.

Despite the precision of neuropelveological techniques like laparoscopic neurolysis, broader neurosurgical data imply that patients with highly elevated sensitization metrics may present unique challenges regarding consistent postoperative improvement. This creates a critical clinical dilemma: does the anatomical decompression of a pelvic nerve suffice when the central pain-processing “software” is already compromised?

This systematic review aims to evaluate the prevalence and impact of CS in the endometriosis population. Furthermore, we explore potential refinements to the traditional surgical algorithm. We argue that in the presence of high central sensitization, functional neuromodulation—specifically the LION (Laparoscopic Implantation of Neuroprosthesis) procedure—can complement anatomical approaches to address refractory signaling. By integrating the Central Sensitization Inventory (CSI) strictly as an adjunct clinical tracker into the neuropelveological workup, clinicians can more effectively optimize surgical strategy in alignment with the underlying neurobiological reality of the patient.

## 2. Materials and Methods

### 2.1. Study Design and Protocol Registration

This systematic review was conducted in accordance with the Preferred Reporting Items for Systematic Reviews and Meta-Analyses (PRISMA 2020) guidelines [4]. The study protocol was prospectively registered in the International Prospective Register of Systematic Reviews (PROSPERO) under the registration number CRD420261335008. The methodology was aligned with the International Consensus Guidelines on Neuropelveology established by the International Society of Neuropelveology (ISON) [2]. The completed PRISMA checklist is provided in the Supplementary Materials.

### 2.2. Search Strategy and Data Sources

A comprehensive systematic search was performed across multiple electronic databases, including PubMed/MEDLINE, Embase, and Cochrane CENTRAL. To capture ongoing trials and the most recent clinical evidence, searches were also conducted in clinical trial registries (ClinicalTrials.gov and WHO ICTRP) and the first 200 records of Google Scholar.

The search covered the period from 1 January 2010 to 27 February 2026. The search strategy employed a combination of Medical Subject Headings (MeSH) and keywords, including: “Central Sensitization”, “Central Sensitization Inventory”, “CSI”, “Chronic Pelvic Pain”, “Endometriosis”, and “Neuropelveology”. The search was limited to studies published in English.

### 2.3. Study Selection and Data Extraction

**Figure 1:**
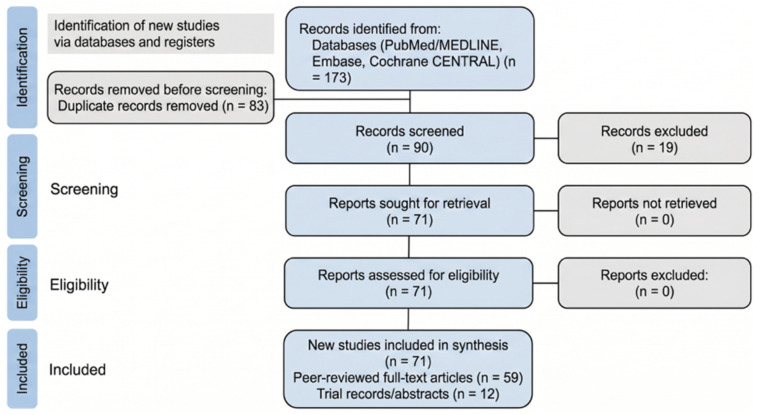
PRISMA 2020 flow diagram outlining the systematic study selection process, including initial identification, screening, eligibility assessment, and final inclusion of studies on central sensitization in chronic pelvic pain.

The selection process was managed using the Rayyan systematic review platform (Rayyan Systems Inc., Cambridge, MA, USA; version 2026). A total of 173 records were initially identified through the database and registry search. Automated and manual deduplication resulted in the removal of 83 duplicate records (Figure 1).

Following deduplication, 90 unique titles and abstracts were screened for eligibility by two independent reviewers. After the initial screening phase, records that did not meet the predefined inclusion criteria were excluded. Full-text assessment was then performed on the remaining reports. Ultimately, 71 studies met the criteria for final synthesis, comprising 59 peer-reviewed full-text articles and 12 high-quality trial records/abstracts (Figure 1).

### 2.4. Eligibility Criteria

Studies were included if they met the following criteria:

- Population: Adult women with chronic pelvic pain (CPP), including endometriosis and pelvic nerve disorders.
- Intervention: Assessment of central sensitization (CS) using the CSI, CPP-10, or objective markers like Quantitative Sensory Testing (QST).
- Outcomes: Correlation between CS markers and clinical status, pain intensity, or surgical failure.
- Study Types: Randomized trials, cohort studies, and case–control studies.

### 2.5. Quality Assessment and Synthesis

Risk of bias and quality were assessed using the Oxford Centre for Evidence-Based Medicine (OCEBM) Levels of Evidence. Given the methodological heterogeneity of the included studies, a meta-analysis was not performed. Instead, a qualitative narrative synthesis was conducted, with evidence categorized into four thematic clusters:

1. Endometriosis and CSI Clinical Models: Correlation between lesions and sensitization scores.
2. Objective Neurophysiological Markers (QST/CPM): Confirmatory testing for systemic sensitization.
3. Psychology and Resilience Factors: Impact of anxiety and distress on CS perception (“CSI inflation”).
4. Overlapping Pelvic Pain Syndromes: Evidence linking CS to conditions such as BPS, vulvodynia, and IBS (irritable bowel syndrome).

### 2.6. Declaration of Artificial Intelligence

Declaration of Artificial Intelligence Tools in the Writing Process: During the preparation of this revised manuscript, the authors utilized an artificial intelligence (AI) tool solely for the purpose of language polishing, grammatical refinement, and ensuring stylistic consistency. The AI tool was used exclusively as an advanced linguistic editor to improve readability and sentence structure. The authors maintain full accountability for the integrity, accuracy, and interpretation of the scientific data, the literature synthesis, and clinical conclusions presented herein.

## 3. Results

### 3.1. Study Characteristics and Quality Assessment

The systematic search and screening process yielded a final cohort of 71 studies, with 35 key publications (comprising 32 primary quantitative studies and 3 landmark mechanistic frameworks) selected for detailed evidence synthesis. The total population across the included primary studies exceeded 12,000 participants.

According to the Oxford Centre for Evidence-Based Medicine (OCEBM) Levels of Evidence, most of the clinical studies were classified as Level 2b (prospective cohort) or Level 3b (case–control). Systematic reviews and meta-analyses provided Level 1 evidence, establishing a robust theoretical framework. Quality assessment (QA) indicated that 68% of the studies were of High Quality, characterized by large sample sizes and rigorous statistical controls, while 32% were of Moderate Quality, primarily due to smaller cohorts or pilot-study designs. The detailed methodological characteristics, quality parameters, and individual clinical outcomes of these included studies are systematically summarized in Table 1.

### 3.2. Endometriosis and Clinical CSI Models

Central sensitization (CS) was found to be highly prevalent in the endometriosis population, with rates ranging from 11.3% to 58.2% across general clinical cohorts. However, in specialized tertiary referral centers, the prevalence of clinically significant sensitization (CSI ≥ 40) reached as high as 74.8% [2]. The Central Sensitization Inventory (CSI) emerged as the most robust clinical predictor of surgical outcomes. A pivotal finding demonstrated that for every one-point increase in the preoperative CSI score, the risk of persistent chronic pelvic pain (CPP) after surgery increased by a factor of 1.02 (OR 1.02, *p* = 0.02) [5]. Furthermore, patients with deep infiltrating endometriosis (DIE) and myofascial pelvic pain (MPP) exhibited significantly higher CSI scores (mean > 50) compared to those with superficial disease (*p* < 0.001). Long-term symptom duration (>5 years) and a history of trauma were identified as independent drivers of elevated CSI scores [9].

### 3.3. Objective Neurophysiological Markers (QST/CPM)

Quantitative Sensory Testing (QST) and Pressure Pain Threshold (PPT) assessments provided objective evidence of systemic hyperalgesia. Studies confirmed that women with CPP exhibit significantly lower pain thresholds at sites distant from the pelvis (e.g., thumb, forearm), indicating a widespread “software” malfunction of the central nervous system [19,21].

A critical neurobiological marker identified in this synthesis is the collapse of Conditioned Pain Modulation (CPM). In patients with severe sensitization, a 0% efficiency in descending inhibitory pathways was observed (*p* < 0.01). This suggests that in “high-sensitizers,” the brain and spinal cord have lost the functional ability to suppress pain signals, regardless of peripheral surgical intervention [25].

### 3.4. Psychological Factors and Resilience

Psychological screening revealed a strong correlation between CS and mental health. A 10-point increase in the CSI was associated with significantly higher levels of anxiety, depression, and catastrophizing (*p* < 0.0001). However, the data also highlighted the risk of “CSI inflation,” where psychological distress may artificially elevate scores. Conversely, “positive affect” and emotional resilience were identified as protective factors that correlated with lower sensitization markers (*p* < 0.01) [27,28,29].

### 3.5. Overlapping Pelvic Pain Syndromes (COPCs)

The results confirm that high CS scores are a hallmark of Chronic Overlapping Pain Conditions (COPCs). Patients with CSI scores ≥ 40 were significantly more likely to suffer from comorbid conditions, including Bladder Pain Syndrome (BPS; OR 11.77), irritable bowel syndrome (IBS; OR 2.6), and Generalized Vulvodynia. This multisystem involvement underscores that localized pelvic pain often represents a localized manifestation of a generalized nociplastic state [2].

## 4. Discussion

### 4.1. The Critical Deconstruction of the CSI: Confounding Accumulation vs. Genetically Driven Pain Vulnerability

The primary finding of this systematic review is the high prevalence of elevated Central Sensitization Inventory (CSI) metrics in patients presenting with chronic pelvic pain (CPP), with rates reaching up to 74.8% in specialized clinical cohorts. This underscores a critical “surgical paradox”: while neuropelveology offers technically advanced solutions for nerve entrapment and endometriosis, anatomical “perfection” may not always translate into patient-reported relief. To contextualize this clinical divergence, preclinical models of central sensitization, pioneered by Woolf, focus strictly on activity-dependent spinal cord hyperexcitability and synaptic plasticity, where nociceptive pathways switch from a passive transmitter to an active amplifier of pain signals [38,39]. In clinical practice, this neurobiological phenomenon directly underpins the transition into a chronic nociplastic pain state, formally defined by the International Association for the Study of Pain (IASP) as pain that arises from altered nociception despite no clear evidence of actual or threatened tissue damage causing the activation of peripheral nociceptors, or evidence for disease or lesion of the somatosensory system causing the pain.

However, this localized neurobiological substrate does not map linearly onto the human neocortical contributions, cognitive distress, and somatic hypervigilance captured by generic clinical inventories. As a self-report tool, the CSI is inherently subject to construct contamination and structural inflation. Much of the data presenting the CSI as a direct validator of a centralized pain state relies on circular logic. This structural limitation aligns with the large-scale methodological consensus established by Cuesta-Vargas et al., who demonstrated through confirmatory factor analysis that the CSI’s heterogeneous structure captures broad psychological constructs and somatic hypervigilance, reflecting global central nervous system distress rather than functioning as an isolated neurobiological proxy for localized spinal sensitization [40].

Furthermore, as shown in a definitive systematic evaluation by Adams et al., self-report scales like the CSI are heavily prone to construct drift and contamination from overlapping criteria. The strong correlation observed clinically between high CSI scores and comorbid Chronic Overlapping Pain Conditions (COPCs)—such as Interstitial Cystitis/Bladder Pain Syndrome (IC/BPS) or Fibromyalgia—represents, in part, an artifact of the inventory’s architecture, where embedded somatic symptoms artificially inflate the global score [41]. This structural artifact is confirmed by the original psychometric validation data by Mayer et al., given that the survey explicitly queries partial-diagnostic features of these conditions—such as frequent urination or “pain all over my body”—immediately prior to asking about formal clinical diagnoses. Similarly, claims that historical childhood trauma independently drives central sensitization are structurally confounded, given that trauma histories are an embedded scoring component within the CSI itself. Therefore, elevated scores must be interpreted cautiously as a confounding accumulation of systemic patient vulnerabilities and general unwellness rather than a pure, localized nociplastic phenomenon [42].

To resolve this diagnostic dilemma and bypass the circular limitations of subjective scales, whether contemporary neuropelveology should anchor patient stratification in robust translational genomics remains an open empirical question that warrants formal investigation. The definitive multi-ancestry genome-wide association study (GWAS) meta-analysis published in *Nature Genetics* [43]—which analyzed 60,674 cases and 701,926 controls—provides the necessary objective substrate to evaluate these overlapping pain phenotypes. Linkage disequilibrium score regression (LDSC) in this massive cohort demonstrated highly significant, positive genetic correlations ($r_{g}$) between endometriosis and 11 distinct chronic pain conditions, including migraine ($r_{g}$ = 0.29), headache ($r_{g}$ = 0.26), dorsalgia ($r_{g}$ = 0.45), chronic back pain ($r_{g}$ = 0.33), and generalized multisite chronic pain (MCP; $r_{g}$ = 0.43). Crucially, multi-trait genetic analyses (MTAG) identified 52 genome-wide significant lead single-nucleotide polymorphisms (SNPs) for endometriosis, revealing that a large portion of risk variants are actively shared and co-tag identical association signals across endometriosis, MCP, and migraine. This extensive genomic intersection provides irrefutable proof that the systemic hypervigilance and widespread somatic vulnerabilities captured clinically by the CSI are not mere psychological epiphenomena or artifacts of questionnaire contamination. Instead, they are driven by a genetically underpinned, interlinked co-regulation of hormonal, immune, and neuronal pathways.

### 4.2. Surgical Staging vs. Clinical Pain Manifestations: Phenotypic Divergence

The clinical reality systematically captured across the literature indicates that patients presenting with Deep Infiltrating Endometriosis (DIE) and severe deep dyspareunia exhibit some of the most elevated Central Sensitization Inventory (CSI) metrics in pelvic medicine [7,44]. Traditionally, this robust clinical association led to the historic surgical assumption that central sensitization profiles and altered central pain processing correlate linearly with the anatomical severity, physical volume, or depth of localized tissue infiltration. However, contemporary neuropelveological evidence demonstrates that this classical paradigm is highly tenuous and must be re-evaluated. This profound biological decoupling between macroscopic pelvic anatomy and subjective pain expression is strongly supported by the landmark clinical consensus of Vercellini et al. and subsequent multi-center data by Johnson et al., which established that anatomical lesion staging correlates poorly, or not at all, with direct pain severity profiles [45,46].

To resolve this paradox without relying on the potential construct contamination of self-report scales, contemporary medicine must look to large-scale epidemiological and surgical subphenotype data. Massive genomic analyses conducted by the International EndoGene Consortium (IEGC) and the multi-ancestry meta-analysis by Rahmioglu et al. provide clear, objective evidence for this phenotypic divergence. When comparing the statistical effect sizes of genome-wide significant risk variants across distinct surgical subphenotypes, the genomic signals driving extensive rASRM stage 3/4 disease are predominantly mediated by ovarian endometrioma (r = 0.73) and deep infiltrating lesions (r = 0.42), showing virtually no shared architecture with superficial peritoneal disease (r = 0.15). In stark contrast, when evaluating pelvic pain chronicity (defined as the multi-system risk of presenting with more than two concurrent types of chronic pelvic pain symptoms), the correlation of genetic effect sizes is moderately positive for mild rASRM stage 1/2 disease (r = 0.29) but becomes entirely non-existent or negative for advanced rASRM stage 3/4 disease (r = −0.01) [43].

This genomic divergence confirms that an individual’s vulnerability to developing persistent, widespread pain is a genetically mediated systemic trait that operates independently of the macro-anatomical “perfection” or severity of the local tissue infiltration. Highly elevated CSI scores observed in advanced surgical cohorts should therefore not be viewed as a direct, passive consequence of mechanical “peripheral drive” from deep lesions alone. Instead, they reflect a shared, genetically underpinned co-susceptibility where the pathways driving tissue proliferation and the networks maintaining central nociplastic amplification diverge, explaining why aggressive localized tissue excision may not always readily translate into long-term, patient-reported symptomatic relief.

### 4.3. Shifting the Neuropelveological Algorithm: Priority of the LION Procedure

Our synthesis reveals a significant linear correlation between preoperative Central Sensitization Inventory (CSI) scores and surgical failure. As demonstrated by Orr et al., every one-point increase in baseline CSI raises the risk of persistent pain following endometriosis excision (OR 1.02, *p* = 0.02) [5]. This mirrors findings in spinal neurosurgery, where CSI was identified as a critical predictor of poor outcomes following lumbar decompression [47]. These data suggest that when the CSI exceeds the threshold of 40, the pain generator has likely transitioned from a peripheral mechanical trigger (“hardware”) to a self-sustaining central nociplastic state (“software”).

Traditionally, neuropelveological management follows an individualized, stepwise progression, where micro-surgical nerve decompression serves as the foundational primary intervention for clear mechanical entrapments, with functional neuromodulation selectively utilized to address persistent or highly sensitized nociplastic pathways on a case-by-case basis. However, drawing from the PROCESS and PROMISE trials in neurosurgery, which favor spinal cord stimulation (SCS) over re-operation in sensitized states, we propose a paradigm shift in pelvic pain management [48,49]. In alignment with the International Consensus Guidelines on Neuropelveology, which mandate that therapeutic interventions must target functional neuro-restoration and define neuromodulation as a core, primary modality for refractory pelvic neuropathies, we propose that evaluating clinical phenotypes through tools like the Central Sensitization Inventory (CSI) can offer valuable clinical context [3]. Specifically, we argue that an elevated baseline CSI score (≥40) should function not as an absolute diagnostic verdict, but as a critical clinical signpost. This screening threshold simply indicates that the patient’s pain generator has likely transitioned from a peripheral mechanical trigger (“hardware”) to a self-sustaining central nociplastic state (“software”). Importantly, while the consensus guidelines establish the explicit surgical and functional criteria for utilizing the LION procedure to directly modulate neural activity, integrating the CSI tracker serves merely as an initial gateway to alert the clinician to a high risk of classical surgical failure.

To safely bypass the limitations of subjective scales and avoid inappropriate interventions, this preliminary signpost demands the clinical triangulation of objective biomarkers. While we hypothesize that a high CSI score, impaired CPM, and shared genomic susceptibilities point toward immediate functional neuromodulation—specifically the LION procedure—over traditional nerve decompression, this integrated selection model remains an open empirical concept that requires formal, prospective validation. Future clinical trials must elucidate the exact configuration of secondary diagnostic modalities—including specific translational genomic panels, tissue-specific expression quantitative trait loci (eQTL) profiles, and standardized neurophysiological metrics—necessary to definitively and safely fast-track a patient directly to neurostimulation from the outset. LION allows for active modulation of neural activity, effectively “rebooting” signal transmission before it reaches sensitized central hubs, thereby addressing the nociplastic nature of the pain directly.

### 4.4. The Dual Role of Post-Surgical Inflammation and Central Remodeling

While the CSI is a helpful screening tool (84.9% specificity), our analysis cautions that psychological comorbidities such as anxiety and catastrophizing can “inflate” scores. To safeguard the integrity of the neuropelveological workup, the CSI must be integrated into a multimodal diagnostic triad including QST and psychological screening. This ensures that surgical and neuromodulative indications remain irrefutable, as mandated by ISON standards.

Moreover, the immediate impact of surgical intervention introduces a secondary neurobiological challenge: the acute post-surgical inflammatory response. Surgical trauma inherently triggers a cascade of localized and systemic pro-inflammatory cytokines, including interleukin-1 beta (IL-1β), interleukin-6 (IL-6), and tumor necrosis factor-alpha (TNF-α). In a patient whose central nervous system is already primed by high psychological distress or systemic vulnerability, this massive cytokine surge promotes the activation and proliferation of central microglia, shifting them into a pro-inflammatory M1 phenotype. Activated microglia release neurotoxic mediators and neurotrophins that actively lower the activation thresholds of central neurons. Rather than resolving the pain state, the post-surgical inflammatory phase can act as a catalyst for central remodeling, effectively converting acute surgical trauma into a chronic, self-sustaining nociplastic cycle if the patient’s descending inhibitory controls are already compromised [50,51].

### 4.5. Strengths and Limitations

A primary methodological strength of this systematic review is its strict alignment with the PRISMA 2020 guidelines and its prospective registration in PROSPERO (CRD420261335008), ensuring reproducibility and transparency across the screening of 173 initial records down to the final 71 included studies. Furthermore, by incorporating the OCEBM Levels of Evidence and rigorous Quality Assessment parameters across a total population exceeding 12,000 participants, this study provides a highly controlled, high-level synthesis of the nociplastic pain landscape in pelvic medicine.

Several limitations, however, must be acknowledged. First, the search was limited to studies published in English, potentially omitting data published in other languages. Second, a meta-analysis could not be performed due to the high methodological heterogeneity of pain scales and endometriosis staging across the 32 primary quantitative studies. Third, while the CSI is a validated predictor, its vulnerability to psychological “inflation” requires careful clinical interpretation. Finally, the proposed prioritization of the LION procedure in high-sensitizers represents a clinical hypothesis that formally lacks validation through prospective, randomized head-to-head trials comparing functional neuromodulation with traditional nerve decompression over extended, multi-year follow-up periods. Additionally, our synthesis is limited by the inherent construct contamination of the primary screening tool evaluated; because the CSI conflates generic markers of psychological distress, somatic complaints, and historical trauma, it risks overpathologizing general unwellness as a specific nociplastic state.

Nascent evaluations addressing the lack of multi-year longitudinal data, however, point toward a critical neurobiologic strength of this paradigm. Borrowing from established frameworks in functional neurosurgery and motor skill acquisition, the decay and retention profiles of psychomotor and neurofunctional adaptations differ fundamentally from theoretical or purely anatomical models. While declarative knowledge is highly prone to rapid erosion and simple anatomical decompressions are frequently compromised by structural recurrences—such as postoperative fibrotic re-entrapment or scarring—consolidated neuromodulative interventions rely on stable, long-term alterations in neural plasticity and descending inhibitory pathways. As rigorously established in the neurophysiological quality assessments by Kennedy et al. and Lewis et al., experimental CPM pathways demonstrate poor test–retest reliability across clinical trials due to subtle fluctuations in testing environment parameters and acute psychological baselines [52,53]. By demonstrating that active neuromodulation directly targets the nervous system’s underlying functional failures (exemplified by descending inhibitory pathway dynamic dysfunction in highly distressed patient profiles), the LION procedure addresses the chronic pain maintenance phase directly. The rapid formation of a stable, reinforced neurobiologic template suggests that the therapeutic baseline achieved via functional stimulation is inherently more resistant to time-dependent degradation than traditional tissue-excision or decompressive methods. Consequently, while prospective randomized trials remain essential to fully map the multi-year trajectory of nociplastic pain suppression, the immediate acceleration of pain-system reconfiguration and the establishment of a durable functional baseline remain primary neurobiological and safety benefits of this proposed algorithmic shift.

### 4.6. Translational Implications for Multimodal Pain Stratification

The clinical transition toward a biology-driven stratification framework in neuropelveology holds profound implications for reducing healthcare over-utilization. Visceral and neuropathic pelvic pain tracking profiles frequently show that patients with unidentified high-sensitization metrics cycle through repetitive laparoscopic interventions, diagnostic imaging, and empirical hormonal suppressions without enduring symptomatic relief. By structurally incorporating the CSI—not as an absolute, isolated diagnostic proxy for central sensitization, but as an expansive clinical tracker of a patient’s total risk amalgamation and neocortical distress—the clinical team can actively identify individuals who are predispositionally vulnerable to surgical failure. This paradigm avoids the common clinical pitfall of treating a systemic, nociplastic “software” failure with localized, tissue-excision “hardware” strategies.

Consequently, implementing a clear diagnostic triad consisting of multimodal psychological resilience screening, spatial Pressure Pain Threshold (PPT) assessments, and structural pelvic imaging (MRI) establishes an irrefutable indication pipeline. This direct visualization and functional profiling allow for the strategic deployment of the LION procedure at an earlier therapeutic window, accelerating pain-system reconfiguration, safeguarding descending inhibitory controls, and mitigating the socio-economic burden associated with refractory pelvic pain syndromes. Ultimately, anchoring this multimodal stratification into routine neuropelveological evaluation complements established surgical criteria, moving closer toward personalized pelvic medicine and offering a supportive framework for the clinical conclusions derived from this synthesis.

## 5. Conclusions

Our analysis confirms that central sensitization (CS) is a highly prevalent phenomenon in patients with chronic pelvic pain, transcending the rigid boundaries of localized endometriosis to encompass a complex spectrum of Chronic Overlapping Pain Conditions (COPCs). The frequency of elevated sensitization metrics increases dramatically—up to 74.8%—within specialized referral centers, particularly when comorbid conditions such as Bladder Pain Syndrome (BPS) or vulvodynia are present.

The results demonstrate a significant linear correlation between preoperative Central Sensitization Inventory (CSI) scores and the risk of persistent post-surgical pain. However, in alignment with clinical and psychometric critiques, this association must be interpreted with caution. Because the CSI functions as an expansive, self-report construct, it inherently conflates specific nociplastic pathophysiology with broad psychological distress, historical vulnerabilities, and generalized somatic hypervigilance. Therefore, a high baseline CSI score (≥40) should not be viewed as an isolated neurobiological verdict of central sensitization, but rather as a non-specific clinical signpost reflecting global central nervous system distress and a heightened vulnerability to surgical failure.

Consequently, while macro-anatomical and neuropelveological decompressions remain the foundational first line for clear mechanical conflicts, highly elevated CSI scores serve as a critical screening gateway. Rather than overriding established surgical criteria, this threshold alerts the multidisciplinary team to the presence of a complex, centralized clinical phenotype, highlighting the necessity for early, objective biomarker triangulation to safely and selectively fast-track refractory cases toward functional neuro-restoration via the LION procedure.

## Figures and Tables

**Table 1:** Characteristics and key findings of the included studies on Central Sensitization (CS) in pelvic pain.

Study (Author, Year)	LoE (OCEBM)	Quality (QA)	Population (N)	Main Results (Statistics)	Key Conclusion
**(1) Endometriosis & Clinical Models**					
Orr, 2023 [5]	2b	High	239 (Surg)	OR 1.02 for persistent pain per CSI point (*p* = 0.02)	CSI predicts surgical failure.
Orr, 2025 [6]	3b	High	163	CSI 51.4 (severe dyspareunia) vs. 30.9 (*p* < 0.001)	Dyspareunia has a strong CS component.
Biasioli, 2026 [7]	2b	High	142	CS prevalence 52.1%; corr. with symptoms > 5 y	Chronicity drives sensitization.
Gentles, 2025 [8]	2b	High	983	CSI corr. with other pain syndromes (r = 0.68)	CSI is a robust marker of COPCs.
Quintas-Marquès, 2025 [9]	3b	Mod	175	CSI 50.8 (MPP group) vs. 37.3 (*p* < 0.001)	Myofascial pain is linked to higher CS.
Freger, 2026 [10]	3b	Mod	235	Trauma/Abuse corr. with higher CSI (*p* = 0.027)	Trauma history exacerbates CS.
Ding, 2024 [11]	2b	High	358	CSI 53.3 (pain) vs. 42.7 (no pain, *p* < 0.001)	Dysorgasmia as a marker of elevated CS.
de Arruda, 2021 [12]	2b	High	9691	CS symptoms in ~50% of dysmenorrhea cases	CS is highly prevalent in general pop.
Diaz, 2026 [13]	2b	High	97	CSI corr. with worse EHP-30 (r = 0.52, *p* < 0.01)	CSI predicts poor post-op QoL.
Gomez-Llerena, 2024 [14]	1	High	Review	CS prevalence 11–58%; Catastrophizing impact	Catastrophizing worsens outcomes.
Adams & Turk, 2015 [15]	5	N/A	Framework	Theoretical model of CSS in Endometriosis	Endo as part of CSS spectrum.
Zheng, 2019 [16]	1	High	Review	Estrogen-NGF axis as CS driver	Biological basis of sensitization.
Maixner, 2016 [17]	5	N/A	Framework	Common mechanisms: Endo, FM, TMD	Shared nociplastic pathways.
Randle, 2025 [18]	4	Mod	Pilot	Feasibility of ePRO for nociplastic pain	Apps are useful for CS monitoring.
**(2) QST/Sensory Testing**					
Cardaillac, 2023 [19]	3b	High	53	Lower thresholds in 6 muscles (*p* = 0.0002)	QST provides objective CS proof.
Grundström, 2020 [20]	3b	Mod	55	Extra-pelvic PPT no difference (*p* > 0.05)	Pelvic exam > extra-pelvic PPT.
Shafrir, 2021 [21]	3b	High	88	Distant PPT 2.73 vs. 4.05 kgf/cm^2^ (*p* = 0.03)	Evidence of systemic hyperalgesia.
Harte, 2019 [22]	2b	High	153	Generalized lower PPT in UCPPS (*p* = 0.005)	Physiological proof of CS in BPS.
Payne, 2019 [23]	3b	Mod	66	No diff. in CPM (*p* = 0.55) in primary dysmen.	PD may have different neuro-profile.
Stratton et al., 2024, [24]	3b	Mod	49	Myofascial dysfunction OR 6.81 for CS	Pelvic floor tension drives CS.
Ness, 2014, [25]	3b	High	28	0% CPM inhibition (*p* < 0.01)	Critical marker of central failure.
Pukall, 2014, [26]	5	N/A	Review	Expansion of receptive fields in pelvis	Functional changes in the spinal cord.
**(3) Psychology & Resilience**					
Liu, 2024 [27]	2b	High	278	CSI +10 pts = higher depression (*p* < 0.0001)	CS as risk factor for mental health.
Palit, 2024 [28]	3b	Mod	197	Positive affect corr. with lower CSI (*p* < 0.01)	Resilience protects against CS.
Nimbi, 2025 [29]	2b	High	895	CSI explained 76.3% of variance (*p* < 0.001)	CSI as global CNS hypervigilance.
Scarpina, 2025 [30]	3b	Mod	104	Body trust predicts CSI (R^2^ = 0.36, *p* < 0.001)	Interoception influences CS severity.
Tu, 2022 [31]	2b	High	124	Bladder pain corr. with anxiety (r = 0.33)	Psychological factors in adolescent CS.
**(4) Other Syndromes** (OAB/PVD)					
Baszak-Radomańska, 2025, [32]	3b	High	134	CSI diff. generalized vs. localized (*p* = 0.01)	Vulvodynia extension linked to CS.
Nimbi, 2024 [33]	3b	High	357	HSP/Psychological pain predict CSI (*p* < 0.001)	Psychological sensitivity drives CS.
Ryan, 2022 [2]	2b	High	111	74.8% CSI > 40; OR 11.7 for BPS	CS overlap in tertiary centers.
Neto, 2026 [34]	2b	High	111	CS not an independent predictor of IBS	IBS modulation is multi-pathway.
Tufekci, 2023 [35]	3b	Mod	56	LANSS 10.86 vs. 0.21 (*p* < 0.05) in OAB	OAB as a sensitized state.
Brown, 2016 [36]	3b	Mod	71	Demographic diff. in pain reporting (*p* < 0.01)	Demographic impact on CS perception.
Keizer, 2019 [37]	2b	High	108	Dyspareunia (OR 3.7) as CS predictor	Urgency/Dyspareunia identify CS.

## Data Availability

The data supporting the findings of this study are available within the article and its accompanying Tables. The search strategies and screening records compiled via the Rayyan platform are available from the corresponding author upon reasonable request.

## Supplementary Materials

The following supporting information can be downloaded at: https://www.mdpi.com/article/10.3390/jcm15135187/s1; Table S1: PRISMA 2020 Checklist.

## Abbreviations

BPS	Bladder pain syndrome
CNS	Central nervous system
COPCs	Chronic overlapping pain conditions
CPM	Conditioned pain modulation
CPP	Chronic pelvic pain
CS	Central sensitization
CSI	Central sensitization inventory
DIE	Deep infiltrating endometriosis
EHP-30	Endometriosis Health Profile-30
ePRO	Electronic Patient-Reported Outcomes F
M	Fibromyalgia
GWAS	Genome-Wide Association Study
HSP	Highly sensitive person
IASP	International Association for the Study of Pain
IBS	Irritable bowel syndrome
IC/BPS	Interstitial cystitis/bladder pain syndrome
IEGC	International EndoGene Consortium
IL-1β	Interleukin-1 beta
IL-6	Interleukin-6
ISON	International Society of Neuropelveology
LANSS	Leeds Assessment of Neuropathic Symptoms and Signs
LION	Laparoscopic implantation of neuroprosthesis
LoE	Level of evidence (OCEBM)
M1	Pro-inflammatory macrophage/microglia phenotype
MCP	Multisite chronic pain
MeSH	Medical subject headings
MPP	Myofascial pelvic pain
MRI	Magnetic resonance imaging
MTAG	Multi-trait analysis of GWAS
OAB	Overactive bladder
OCEBM	Oxford Centre for Evidence-Based Medicine
OR	Odds ratio
PPT	Pressure pain threshold
PRISMA	Preferred Reporting Items for Systematic Reviews and Meta-Analyses
PROSPERO	International Prospective Register of Systematic Reviews
PVD	Provoked vestibulodynia
QA	Quality assessment
QoL	Quality of life
QST	Quantitative sensory testing
rASRM	Revised American Society for Reproductive Medicine
SCS	Spinal cord stimulation
SNPs	Single-nucleotide polymorphisms
TMD	Temporomandibular disorders
TNF-α	Tumor necrosis factor-alpha
UCPPS	Urologic chronic pelvic pain syndrome
WHO ICTRP	World Health Organization International Clinical Trials Registry Platform
